# Impacts of COVID-19 on the Education, Life and Mental Health of Students in Bangladesh

**DOI:** 10.3390/ijerph19020785

**Published:** 2022-01-11

**Authors:** Fahmida Liza Piya, Sumaiya Amin, Anik Das, Muhammad Ashad Kabir

**Affiliations:** 1 Department of Computer Science and Engineering, Chittagong University of Engineering and Technology (CUET), Chattogram 4349, Bangladesh; u1504107@student.cuet.ac.bd; 2 Department of Computer Science, St. Francis Xavier University, Antigonish, NS B2G 2W5, Canada; x2020gae@stfx.ca (S.A.); x2021gmg@stfx.ca (A.D.); 3 Department of Computer Science and Engineering, Bangladesh University, Dhaka 1207, Bangladesh; 4 School of Computing, Mathematics and Engineering, Charles Sturt University, Bathurst, NSW 2795, Australia

**Keywords:** COVID-19, education, mental health, daily life, social life, university students, Bangladesh

## Abstract

COVID-19’s unanticipated consequences have resulted in the extended closure of various educational institutions, causing significant hardship to students. Even though many institutions rapidly transitioned to online education programs, various issues have emerged that are impacting many aspects of students’ lives. An online survey was conducted with students of Bangladesh to understand how COVID-19 impacted their study, social and daily activities, plans, and mental health. A total of 409 Bangladeshi students took part in a survey. As a result of the COVID-19 pandemic, 13.7% of all participants are unable to focus on their studies, up from 1.2% previously. More than half of the participants (54%) have spent more time on social media than previously. We found that 45% of the participants have severe to moderate level depression. In addition, 48.6% of the students are experiencing severe to moderate level anxiety. According to our findings, students’ inability to concentrate on their studies, their increased use of social media and electronic communications, changing sleep hours during the pandemic, increased personal care time, and changes in plans are all correlated with their mental health.

## 1. Introduction

SARS-CoV-2 (severe acute respiratory syndrome coronavirus 2) is a highly contagious and pathogenic coronavirus that appeared in late 2019 [1]. This novel virus has caused an acute respiratory disease pandemic called “Coronavirus Disease 2019” (COVID-19), which endangers public health. On 30 January 2020, the World Health Organization declared the COVID-19 epidemic an international public health emergency, and on 11 March 2020, it was declared a pandemic [2]. As of 26 June 2021, over 180 million people worldwide have been infected by COVID-19, and more than 3.9 million people have died from the disease [3].

In March 2020, the virus was reported to have spread to Bangladesh. On 8 March 2020, the first three cases were registered [4] by the Institute of Epidemiology Disease Control and Research (IEDCR), which is the country’s epidemiology institute [5]. During March, infection levels remained low, but in April, they began to rise markedly [6].

According to [7], 1.3 billion students have been affected by educational institution closures in 186 countries because of COVID-19. Bangladesh is in a similar predicament. On 22 March, Bangladesh’s government proclaimed a 10-day general holiday from 26 March to 4 April on 22 March [8]. Educational institutions, workplaces, and markets were shut down due to restrictions that lasted more than two months. Later, these restrictions were lifted, but they were imposed again after the resurgence of COVID-19 in 2021 [9]. Now, it has been more than a year and the closure of educational institutions is continuing [10].

COVID-19 has taken a heavy toll on students by hampering their academic activities in Bangladesh as well as worldwide. Students cannot physically join regular classes as educational institutions are not permitted to continue on-campus academic activities. Many of the higher educational institutions have moved their academic activities to online platforms after recovering from the initial shock due to the pandemic [11]. However, as many students in Bangladesh come from rural areas where fast internet services are a luxury, they have had to go through significant difficulties. An unwillingness to engage in online education is discussed in [12]. Many of these participants are devastated as they are missing out on an important time of their life.

The changed and disorganized lifestyle of some of the students is impacting their daily life [13] as well as their education [12]. Too much social media usage is hampering their social life balance, which is affecting their overall mental well-being [14]. The authors in [13] discussed the disruptions to the daily life of some office workers along with some undergraduate students from India. They mentioned disrupted health conditions, well-being, and sleep due to daily routine disturbance, anxiety, loneliness, a greater burden on family and work, and excessive screen time.

The COVID-19 pandemic is linked to exceptionally high levels of psychological distress among students [14,15], and it can also create symptoms similar to those seen in post-traumatic stress disorder (PTSD) [16]. A study conducted on French university students focusing on COVID-19 confinement reported an increase in anxiety among two-thirds of the participants [17]. Another study found intensified anxiety, depression, and suicidal thoughts during the lockdown period due to COVID-19 among university students in Greece [18]. A study on college and university students found that, among the respondents, 28.5% had stress, 33.3% had anxiety, and 46.92% had mild to severe depression [19]. Few studies [20,21] have focused on COVID-19 impacts on Bangladesh students’ mental health, but to the best of our knowledge, no study has investigated the correlation among students’ mental health and their education (study hours), social life (including internet and social media usage), daily life (e.g., sleep pattern), and their best and worst experiences during COVID-19.

In this study, an online survey was conducted focusing on Bangladeshi students and the problems and challenges they face in their education, social life, daily life, plans, and mental health. The chi-square test was used to examine the relationship between variables. A heatmap was developed using Cramér’s V to compare the impact of the categorical variables. A word-cloud was used to examine the open-ended questions that the participants answered. To measure their mental health, especially their level of depression and anxiety, PHQ-9 and GAD-7 were used as mental health clinical scales. We also analyzed the relationship between mental health (such as depression and anxiety) and education, social life, day-to-day activities, and plans for the future.

Our study focuses on evidence-based impact analysis, exploring associated factors hampering the mental health of the students of Bangladesh. This work will address the problems that students have to deal with in different aspects of their lives. From these findings, policies can be developed and appropriate actions can be taken by the authorities to support and help students during the COVID-19 pandemic and prepare for post-pandemic life. The students can be provided with appropriate mental or financial support so that they can overcome their difficulties. This paper makes the following five major contributions:

(i) We have analyzed five important aspects of students’ lives, which are their education, social life, daily life, plans for the future, and mental health.
(ii) We have analyzed the mental health of students and found that more than 70% of participants are going through mild to severe depression and anxiety.
(iii) We have assessed the individual correlation among students’ mental health and their education, social life, daily life, and plans for the future.
(iv) We have identified how the mental health of students, their study hours, internet usage, sleep hours, personal care time, and changes in their plans are directly affecting each other during this crucial period.
(v) We have also discovered the reasons behind the changing patterns in students’ study hours, social media usage, and sleep hours and discovered their best and worst experiences during COVID-19.

## 2. Related Work

Several studies have been conducted regarding the impacts of COVID-19 on students’ education. One study, focusing on West Bengal in India, found that 70% of students of that state are involved in online learning due to COVID-19 and were experiencing various issues related to this process [22]. An online survey of college and senior high school students, with 530 respondents, showed that there was some unwillingness to engage in online-blended learning methods [12]. Another study found that the students are not achieving their desired results via online education in a developing country such as Pakistan, as many students do not have reliable internet access due to various technical issues and financial difficulties [23]. Another study on the students of Hanoi, Vietnam, investigated their learning habits during the school suspension due to the pandemic [24]. In a study of Chinese university students, the authors expressed their concern that students from remote and rural areas may lose access to higher education [25] as distance learning is inevitable for the duration of the pandemic [26]. Furthermore, a significant correlation has been observed between educational performance and mental health—the better the mental health of the students, the better their academic performance [27].

Researchers have also focused on the impacts of COVID-19 on students’ social lives. In a study conducted on the general population of Wuhan, China, more than 80% of participants reported frequent exposure to social media during the COVID-19 outbreak [14]. Mental health problems have been found to be positively associated with frequent social media exposure (SME) during the COVID-19 outbreak [14,28]. Another study focused on the association between students’ social media usage and their mental health. It showed that a higher level of social media usage resulted in poor mental health conditions [29]. In addition, the pandemic and associated lockdown increased students’ dependency on different electronic devices to communicate online, resulting in increased screen time [13].

The pandemic has had a significant effect on the daily routines of students worldwide [30]. Researchers have found disruptions in the daily routines of students and corporate job holders due to home confinement and restriction strategies. Furthermore, sleep prolongation and the duration of daytime naps of the students increased compared to daily life before COVID-19 [13]. Younger adults were found to be more concerned about their finances in their daily life [31].

COVID-19 has altered the plans of students worldwide. Many are highly worried about their future [32]. A large-scale survey was conducted to discover how students around the world are dealing with the unpredictable and unprecedented effects of the COVID-19 pandemic, including its negative impact on their current and future lives [30]. Students are worrying about their future employment opportunities “most of the time” or “all of the time” during this pandemic [30].

Numerous studies have focused on mental health-related issues as students are suffering during the COVID-19 pandemic [20,21]. A cross-sectional study that was particularly performed on medical students found that 64.41% of the participants were suffering moderate or severe depression and 46.17% were suffering moderate or severe anxiety [33]. During the pandemic, the mental health of Chinese college students was assessed, providing a theoretical foundation for psychological interventions with college students as well as a framework for national and government policies [32]. The psychological effects of quarantine have been observed in students and others, where authors identified the “fears of getting infected”, “longer duration of quarantine”, “frustration”, “boredom”, “inadequate supplies of necessities”, and “financial difficulties” as the main stress factors [34]. Researchers also explored anxiety, depression level, cognition, and the psychological state of college students in China during the pandemic [35]. Another study revealed that 85.6% of participants were mentally stressed due to COVID-19 [36]. Hampered scheduled study, concern about their future career, a fear of getting infected, and financial insecurities were the key stress factors among students [32]. In addition, as compared to full-time staff, students had substantially higher levels of depression, anxiety, and stress [37].

Researchers disclosed how different sorts of educational and governmental mitigation initiatives showed a minor but long-lasting effect on university students’ mood and wellness behaviors [38]. Several methods are discussed to help students through these tough times [39]; however, there are very few resources in a developing country such as Bangladesh to investigate the effect of COVID-19 on students’ schooling, social lives, everyday lives, plans for the future, and mental health. To the best of our knowledge, although there has been some work on the mental health of Bangladeshi students [20,21], there is no work that considers all these five aspects which cover their education, life, and plans and correlates them with their mental health. Interventions relating to these topics are extremely important, and the government and other responsible agencies must investigate them.

## 3. Methodology

### 3.1. Data Collection Procedure

We performed a cross-sectional study of university students in Bangladesh through an online survey over three months. An online questionnaire was designed for the survey to collect information from the participants. The survey was translated into both English and Bengali so that it was easier for the participants to understand the meaning of the questionnaire. The online survey was conducted using Google forms, and we used different social media and online communication platforms such as Facebook, WhatsApp, Messenger, and Skype to promote the survey among university students. Before participants started the questionnaire, we informed the participants about the study’s basic goals. Furthermore, the consent of every respondent was taken before the survey, and their identity remained anonymous throughout our the study. If a participant did not give their consent or if they were not a university student or a recent graduate (identified through a screening question), the survey ended with a thank you note.

### 3.2. Measures

We prepared the questionnaire addressing five factors: education, social life, daily life, future plans and mental health. In general, the questionnaire was divided into nine sections. The first section was related to the consent statement of the participants. The second section was to confirm that the participants were current students living in Bangladesh. The following section contained questions about the impacts of COVID-19 on education; for example, whether the educational institutions are conducting online classes, whether students have available facilities for online classes, how study hours have been altered during COVID-19, and whether the study focus has been hampered because of the pandemic. The fourth section covered the effects of COVID-19 on students’ social lives, which included questions about the time they spent on social media, online meetings, and internet usage before and after the pandemic. The next section covered questions related to the students’ daily lives, which included questions about sleep duration, enjoyable time spent with family, time devoted to personal care, and alterations in family income along with the cause. An open-ended question was also asked to apprehend the possible causes of changed bedtimes. The next section covered questions regarding student’s plans and which specific factors troubled them most. Here, two open-ended questions were given to identify the best and worst experiences of individual participants during this pandemic.

The research group created the questionnaire, which included open-ended, multiple-choice, and Likert scale-type questions based on applicable literature. Two scales were used in this study to assess students’ mental health, especially their anxiety and depression. Firstly, a nine-item depression scale, the Patient Health Questionnaire-9 (PHQ-9), was used to assess symptoms of depression [40]. The PHQ is a self-administered version of the Primary Care Evaluation of Mental Disorders (PRIME-MD) diagnostic instrument for common mental disorders. It is a useful clinical research tool to identify depressive symptoms. Secondly, the Generalized Anxiety Disorder-7 (GAD-7), was used to assess symptoms of anxiety [41]. GAD-7 is a self-administered patient questionnaire that is used to test for generalized anxiety disorder and to assess its severity. PHQ-9 and GAD-7 scores are determined by assigning scores of 0, 1, 2, and 3 to the response categories of “not at all”, “several days”, “more than half of days”, and “nearly every day”, respectively, and then adding together the scores for the nine questions in case of PHQ-9 and seven questions in case of GAD-7. Several studies have used these scales to screen mental health or to identify the mental well-being of a targeted population [33,42,43,44,45].

### 3.3. Data Analysis

We performed multiple statistical analyses. First, we calculated the mean and standard deviation for both the PHQ-9 and GAD-7 scales. After that, we used the chi-square test to find out if there was any relationship between different factors and the level of depression and anxiety. A *p*-value of less than 0.05 was considered statistically significant. A *p*-value is a metric that expresses the likelihood that an observed difference may have occurred by chance [46]. The statistical significance of the difference observed is proportional to the *p*-value. Then, to depict the correlation between multiple variables, graphical representations were used.

Later, we used a heatmap correlation for graphical representation using Cramér’s V. A heat map is a two-dimensional data visualization technique that displays the magnitude of a phenomenon in color. Cramér’s V is a measure of association between two nominal variables (sometimes referred to as Cramér’s phi). It is also the most common strength test used when a considerable chi-square result has been obtained [47]. Matplotlib [48] is used to plot our categorical heatmap. This is a plotting library of the Python programming language for the development in Python of static, animated, and interactive visualizations.

Finally, for open-ended questions, we used a word cloud [49]. A word cloud is a range of words represented in various sizes or a cluster. The bigger and bolder the word appears, the more frequently it is used, and the more relevant it is in a source text (such as a speech, blog post, or database). The word clouds were generated from our database of students’ answers to the open-ended questions that highlighted their feelings, opinions or perspective.

## 4. Results

In this section, we discuss the noteworthy impacts caused by the pandemic among Bangladeshi students. Firstly, we investigate the impacts of COVID-19 on five aspects of the students’ lives by analyzing the questionnaire. Then, we investigate the correlation between different aspects of the students’ lives with depression and anxiety using the chi-square test. We also generate a heatmap using Cramér’s V to measure the effects among several variables of our five factors. Finally, we examine the open-ended questions answered by the students to measure the best and worst experiences during the pandemic.

Table 1 shows the socio-demographic distribution of the participants from our survey. Among 409 participants, most of the participants were from the Dhaka division. There was a reasonable gender balance, with 63.1% of respondents being male. Most of the participants (71.9%) were aged 18 to 24 years. All the respondents were university students. The largest proportion (79%) of participants belonged to the engineering universities. There were students from public and private universities from Bangladesh and a few students from international universities residing in Bangladesh.

### 4.1. Impact of COVID-19 on Studies

Universities worldwide have moved from on-site to online teaching to minimize the spread of the novel coronavirus [50,51]. In our survey, students were asked if they were receiving online education. The results show that 76% of participants are receiving live online classes regularly, 6.1% are receiving limited online education (i.e., no live classes, only resource sharing and video lecture uploading), and 17.9% said they are not receiving any kind of online education during the pandemic.

The transition of the education system from on-site to online was so quick that there was not much time to properly adapt to the new platforms of education, even though the quality of teaching and learning in these new circumstances needed proper attention [50]. Hence, the students were asked about the basic facilities that were important for conducting fully functional online classes; e.g., whether they had access to a stable internet connection, comfortable places to sit, and a reliable computer or smart device. The responses showed that 60.8% of the students had all the facilities, while 34.2% responded that they had some of these facilities, and 5.1% said they had no facilities at all. Our findings show that the university fees remained unchanged, even though students were neither attending classes physically nor using university facilities. In addition, many of the students were found to be struggling to pay the fees; for some, it is nearly impossible to pay the fees.

Before the pandemic, students spent a lot of time on their studies and other study-related activities [52]. In our study, we found a similar result when we asked the students about their study hours before and during the pandemic. Fifteen percent of the participants did not spend any time (0 h) on study during COVID-19, compared to only 1.4% before the pandemic. However, the study hours of some participants increased during the pandemic. Previously, 2.9% of the participants spent >6 to 7 h, whereas now about 4% are spending this amount of time. Moreover, most of the participants are now spending >7 to 8 or >8 to 9 h on their studies during the pandemic. The reasons we have identified for the changing study times include students finding the online platform inconvenient for studying, meaning that they are still struggling to cope with this new situation. However, students are now spending more time at home as they do not need to travel to their institutions, meaning they can spend more time on their studies.

We also asked the participants how their focus has changed. During COVID-19, 13.7% of the participants cannot concentrate on their studies at any point, compared to only 1.2% previously (before COVID-19). The fast transition to online platforms has made students uncomfortable as this is a very new mode of studying for the students. The drastic changes in the percentages clearly reflect the negative impacts of COVID-19 on the students’ attentiveness.

### 4.2. Impact of COVID-19 on Social Life

The outbreak of COVID-19 has affected the social life of the students. According to [53], people are spending more time on social media networks. People used to spend almost 144 min per day on average on social media [54]. In some countries, people spend more or less than this average time. We divided the students into three categories. More than half of the participants (54%) have been using social media platforms such as Facebook, Messenger, Instagram, Snapchat, WhatsApp, etc. for more than four hours a day. This shows that the internet usage of the students has increased greatly.

The participants provided details about their social media usage, overall internet usage, and the time spent on different online meeting platforms. Online meetings are another addition to the educational life of students in Bangladesh. Among the participants, 73.6% did not spend any time in online meetings per week before COVID-19; this has changed significantly during this pandemic. Now, 22.2% of the participants are spending 0 to 2 h in online meetings every week. In fact, 9.5% of participants reported that they have to spend more than 14 h in online meetings, which is a remarkable impact of this pandemic. One of the reasons is that a significant number of students are receiving online education (i.e., they need to attend online classes, submit assignments, and do exams); therefore, they need to participate in many online meetings constantly.

The students were asked about their overall internet usage, which includes their meeting time, their social media usage, and other website browsing. Before COVID-19, 65.8% participants spent 2 to 6 h using the internet each day, and the proportion during the pandemic is similar (63.8%). However, 31.3% of participants now spend more than 6 h on the internet, compared to only 7.1% before the pandemic.

### 4.3. Impact of COVID-19 on Daily Life

The strong transmission rate of COVID-19 has affected the daily lives of students worldwide [55]. We have tried to identify the changes in the sleep patterns of the participants. The participants are divided into three categories, according to their sleep pattern. The categories have been created according to the recommended sleep pattern in [56], which is 7 to 9 h for young adults and adults and 7 to 8 h for older adults. Before the pandemic, nearly three-quarters of the participants (74.3%) had insufficient sleep. However, now almost half the participants (47.2%) are getting an adequate amount of sleep. In addition, 20.5% of participants are now oversleeping, compared to only 1.5% before the pandemic. This finding shows that many of the participants have increased their allotted time for sleeping.

We have investigated how much time the participants are spending on their personal care; e.g., hobbies, physical exercise, yoga, etc. We have found that more participants (47.4%) are spending at least 2 to 4 h per day on their personal care during the pandemic, compared to 30.8% before the pandemic.

Our study has found that, during this pandemic, students are spending more time with their family. During COVID-19, 37.7% of the participants are spending more than 7 h with their family each day, compared to only 4.6% before COVID-19. As most students are doing their classwork from home and cannot go outside, they are spending more quality time with family.

To analyze the impact of COVID-19 on students’ daily lives, we also observed any change in students’ family income. Among the participants, 76% responded that their family income decreased because of the pandemic, while, only 1.71% experienced an increase in their family income. We further investigated the possible causes of income variance (before and during the pandemic) and found that salary deduction (20%), losing tuition (19.6%), and losing a job (14.9%) were some major impacts of COVID-19 on income source. Only 3.7% of participants have started new jobs during this pandemic to offset the reduction of their family income.

### 4.4. Impact of COVID-19 on Plans for the Future

The world is facing a major crisis due to the spread of the novel coronavirus [25], and based on the previous records of similar events [57], students are very worried about their short and long-term future plans [58]. In our study, 82.9% of the participants responded that their future plans have changed because of COVID-19. Some responded that they have changed their career paths (17.6%) or delayed higher studies (12.7%). Some participants had made multiple changes to their future plans (22.7%). In contrast, 28.6% of participants are now focusing more on skill development, which can help them after the pandemic. Only a few participants (2.4%) also mentioned some other positive plans such as starting their own business online or offline. When asked about their best achievement during this pandemic, some participants identified honing their skills in their related fields or doing or learning something “out of the box” as they now had plenty of spare time.

### 4.5. Impact of COVID-19 on Mental Health

The COVID-19 pandemic has had a major impact on the emotional well-being and mental health of individuals worldwide [34], either specifically in relation to health conditions or indirectly in relation to the economic and social ramifications [30]. Many people have faced intolerable psychological strain, especially because of the impact of the pandemic on everyday life, the economic consequences, and delays in academic activities [32,35]. The mental health of students was affected to varying degrees during the outbreak.

Level of depression among students: The level of depression among the participants was measured according to the PHQ-9 scale. The students were asked about their interest in doing things, if they were feeling down, depressed or hopeless, their sleep quality and timing, their energy levels, appetite, how they were feeling about themselves, if they were having trouble concentrating on different things, if they were moving or speaking slowly or getting restive, and if they ever had any thought of hurting themselves or if they had negative thoughts about their existence. We found that 7.1%, 11.2%, 26.7%, and 28.9% have severe, moderately severe, moderate, and mild depression respectively. Only 26.1% of the total participants were found to have no depressive symptoms. The mean was 9.24 and the standard deviation was 6.163.

Level of anxiety among students: Students have suffered from various degrees of anxiety throughout the pandemic. The level of anxiety was measured by using the GAD-7 scale. The students were asked if they were feeling nervous, anxious or on edge, if they could stop or control worrying, if they worried too much, if they faced any trouble while trying to relax, if they felt too restless to sit, if they became easily annoyed or irritable, and if they felt afraid that something awful could happen. According to our data, among the participants, 12.7%, 35.9%, 29.6%, and 21.8% have severe anxiety, moderate anxiety, mild anxiety, and minimal anxiety, respectively. The mean was 7.74 and the standard deviation was 7.

### 4.6. Dependency Measurement with Mental Health

We used chi-square analysis to identify dependencies between various aspects of a student’s life and their mental health, specifically their depression and anxiety levels (Table 2). The factors we considered for finding links between participants’ mental health and education were online classes, facilities for conducting online classes, study hours, and focus during the pandemic. Then, to find links between their social life and mental health, we considered time spent on social media, online meetings, and overall time spent on the internet. To find the relationship between mental health and daily life, the factors we considered were sleep time, time spent with family, personal care time, family income change, and change in future plans. The significant relationships are further expressed using graphical representations.

After using the chi-square test, significant relationships were found between depression and anxiety levels and other factors, such as students’ focus on their study, their social media usage and overall internet usage, sleep time, personal care time, and changes in future plans (these factors and mental health are dependent on each other). These factors significantly impacted the mental health (e.g., depression and anxiety) of the participants.

**Figure 1:**
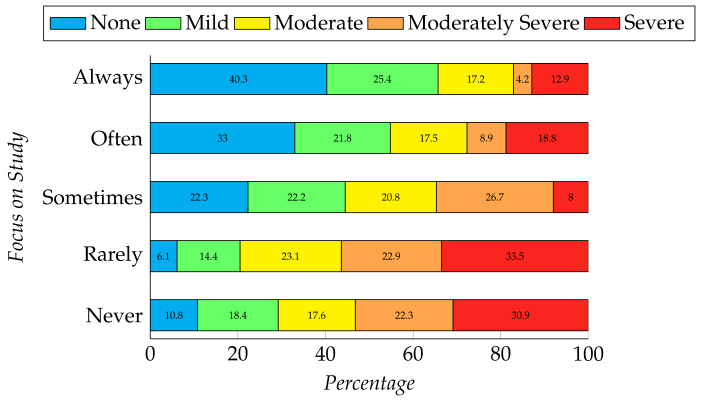
Relation between students’ focus on study and their level of depression.

**Figure 2:**
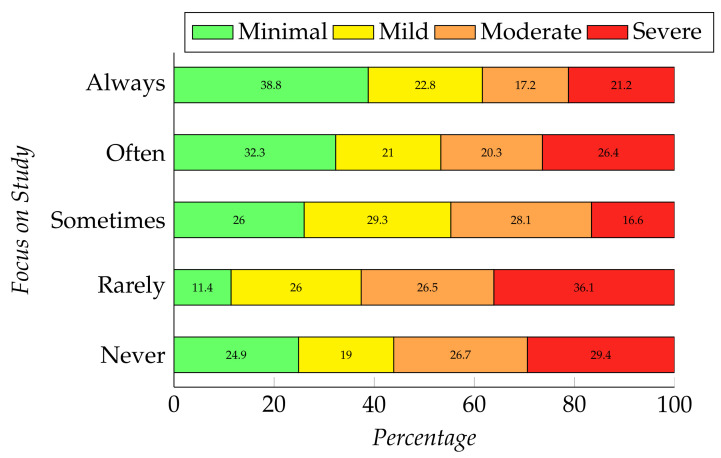
Relation between students’ focus on study and their level of anxiety.

The chi-square result shows a significant relationship between focus on study during the pandemic with both depression and anxiety levels (depression, χ${}^{2}$ (16, N = 409 ) = 50.477, *p* = 0.000 and anxiety, χ${}^{2}$ (12, N = 409 ) = 26.426, *p* = 0.009). Figure 1 and Figure 2 represent the relationship between students’ focus during the pandemic and their mental health; i.e., depression and anxiety, respectively. Here, we have found that the students who can rarely or never focus on their studies are suffering from severe depression (33.5%, 30.9%, respectively) and severe anxiety (36.1%, 29.4%, respectively). Participants who can always concentrate on their studies have no symptoms of depression (40.3%) and a minimal level of anxiety (38.8%).

**Figure 3:**
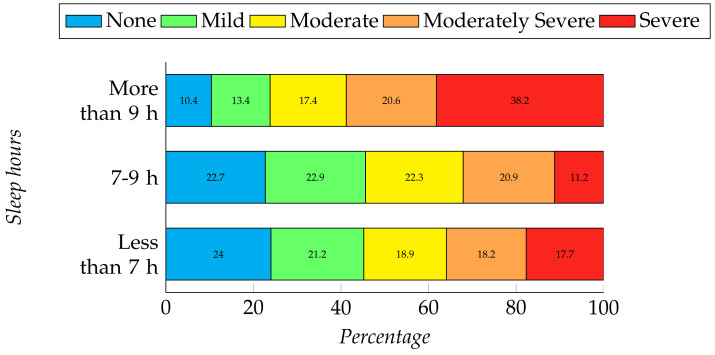
Relation between students’ sleep hours and their level of depression.

We have found a significant relationship between a student’s sleep time and mental health through the chi-square test (depression, χ${}^{2}$ (8, N = 409) = 20.564, *p* = 0.008 and anxiety, χ${}^{2}$ (6, N = 409) = 7.294, *p* = 0.295). The participants who have been sleeping for more than nine hours, which is greater than the recommended average time [56], are suffering from poor mental health. However, the participants who are maintaining the average sleep time, which is 7–9 h per day, are suffering less from depression. The participants who are not having sufficient sleep are also facing some issues of depression (see Figure 3).

**Figure 4:**
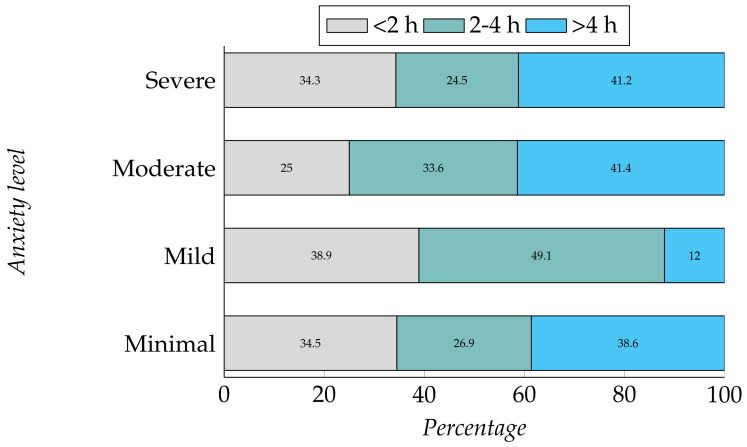
Relation between time spent on personal care and their level of anxiety.

Using the chi-square test, we have found a dependency between the time spent on personal care and the anxiety level of the students (depression, χ${}^{2}$ (8, N = 409) = 8.933, *p* = 0.348 and anxiety, χ${}^{2}$ (6, N = 409) = 14.862, *p* = 0.021). Participants having all levels of anxiety (except mild anxiety) are spending more than 4 h each day on taking care of themselves (see Figure 4). As participants are spending more time at home, they are spending more time than before on personal care. However, we did not find any dependency between participants’ level of depression and personal care.

A strong correlation has been found between students who have changed their future plans and their mental health (depression, χ${}^{2}$ (4, N = 409) = 17.489, *p* = 0.002 and anxiety, χ${}^{2}$ (3, N = 409) = 19.966, *p* = 0.000). Among those participants who have changed their future plans, 22.7% are facing severe depression and 27.4% are facing severe anxiety. On the other hand, the participants who did not need to change their plans are facing a low level of anxiety. Among the participants who have made no changes to their plans, 38.8% have no depression and 47.2% have shown only a minimal level of anxiety.

**Figure 5:**
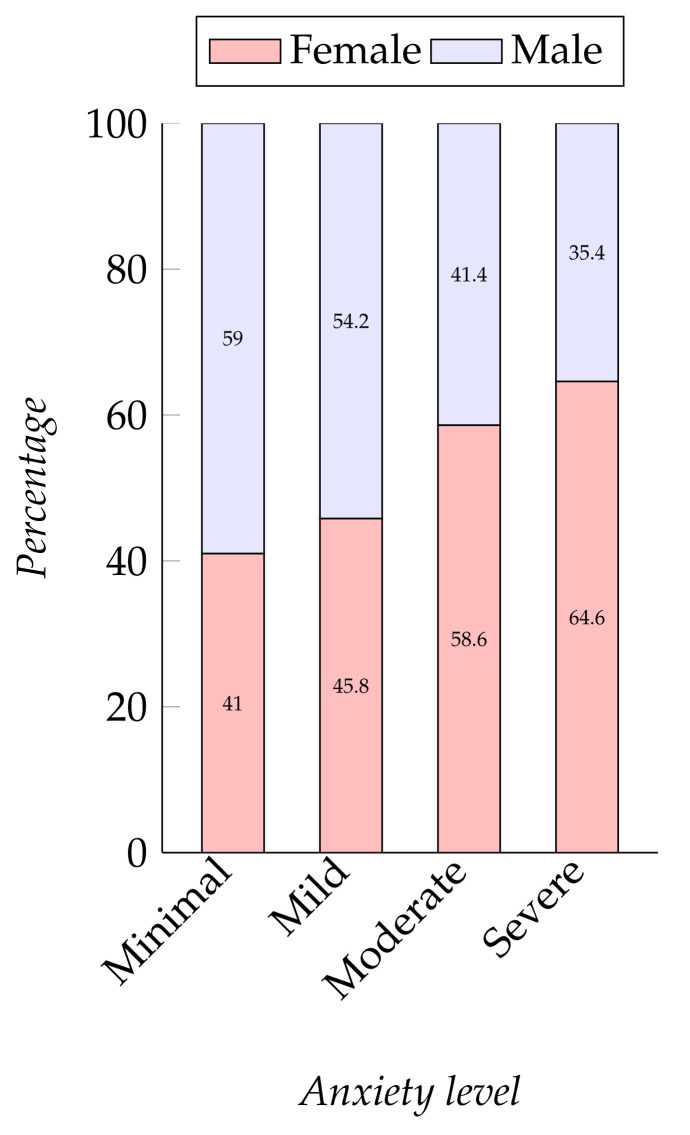
Participants’ gender and their level of anxiety.

A dependent relationship has been found between participants’ gender and their level of depression (depression, χ${}^{2}$ (4, N = 409) = 7.577, p=0.108 and anxiety, χ${}^{2}$ (3, N = 409) = 12.949, p=0.005). Overall, we found that a greater portion of female students had severe depression and anxiety, at 64.7%, which is almost twice the rate for male students (see Figure 5). A review paper [15] examining several other studies on the impact of COVID-19 showed similar findings, with more mental health problems among female participants.

### 4.7. Dependency Measurement for Categorical Variables

We used the result of Cramér’s V to generate a heatmap using different questions from the questionnaire. Every question apart from multiple choice and checkbox option answers was considered in the Cramér’s V result. The values of the variables were stored in an Excel sheet.

**Figure 6:**
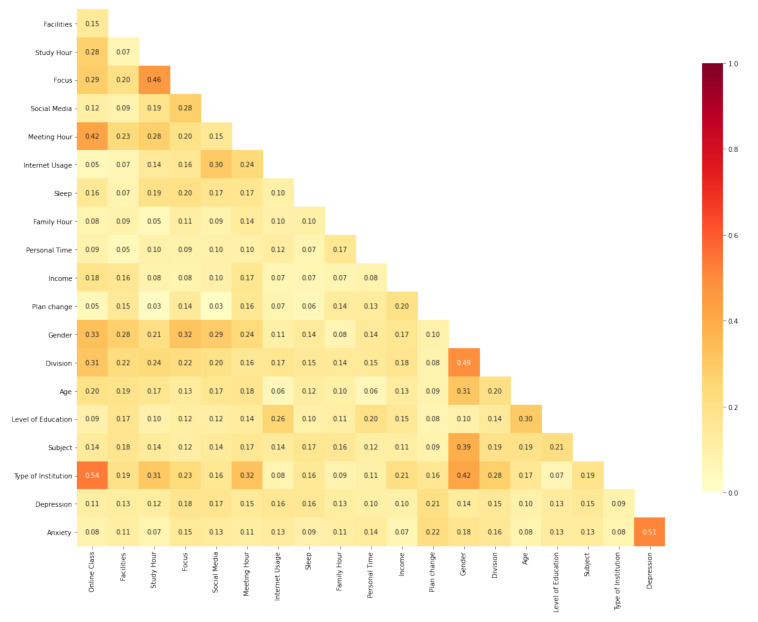
Correlation heatmap.

The graphical illustration was manually created using Python from the Excel spreadsheet and depicts the Cramér’s V value among variables (see Figure 6). The result showed a strong dependency between students’ focus and their study hours. Both the meeting hours and institution type showed a strong relationship with online classes. In addition, depression and anxiety are strongly related to each other.

There are similarities between the results of Cramér’s V and the, chi-square test. The factors that were found to be dependent on each other using the chi square test also showed a significant Cramér’s V value (>0.10). However, for the limitations of Cramér’s V, which is the tendency of generating a relatively low correlation value for highly significant outcomes [47], Cramér’s V generated a low dependency value even though the chi-square dependent factors had high significance (lower *p* value).

### 4.8. Open-Ended Question Analysis

The participants were asked five open-ended questions in which they shared their thoughts, feelings or opinions. They were asked about the reasons behind any changes to their study hours. Most of them mentioned irregularity in their routine, changed study patterns, and changes in class schedule. For some, their study hours have increased as they do not need to travel anymore. Thus, they have saved a great deal of time and can invest in study-related activities.

Participants identified the reasons for their changing pattern of internet usage as follows: (1) daily online classes, (2) online viva, (3) mandatory presence in an active online group, (4) compulsory connection to course teachers, and (5) online group study. One of the participants said, “*Due to the pandemic, all my works have been shifted to online. Again, as I cannot go out in this situation, I am spending most of my time browsing the internet both for recreational activity and work. That’s why my internet usage has increased during COVID-19*”.

Many of the participants also experienced significant changes in their sleep hours. Most of the participants mentioned that they could sleep more as they do not need to attend early morning classes. Some of them have fewer tasks to do, so they are spending more time sleeping.

**Figure 7:**
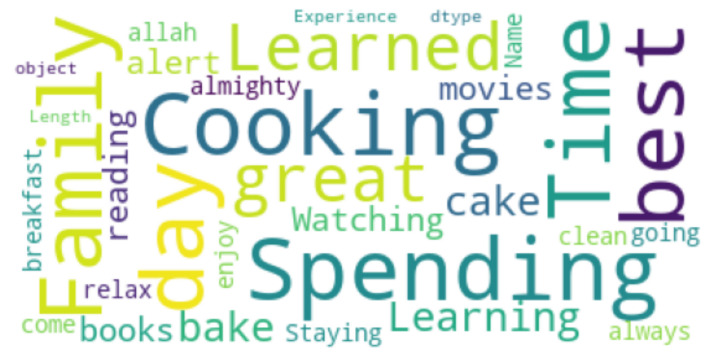
Word cloud representation of best experience.

**Figure 8:**
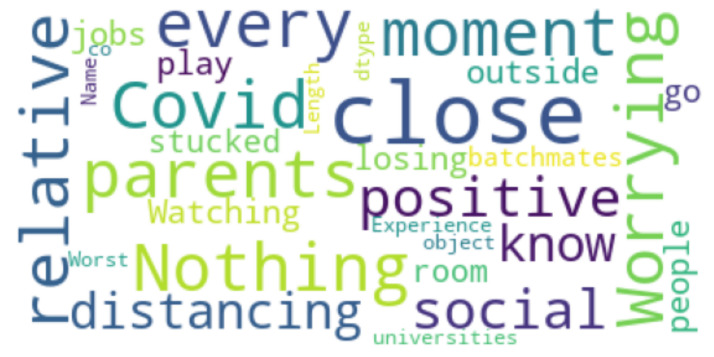
Word cloud representation of worst experience.

The participants were also asked about their best and worst experiences during the pandemic. We generated two word clouds to visualize the answers (see Figure 7 and Figure 8). Many of the students disclosed that the positive experiences included more time spent with family and more free time to do their favorite things, and many of them spent more time on different things such as cooking and reading. Furthermore, many of the students are now focusing on their skills and career development. The students can also spend some time on personal care, including exercising and hobbies, etc. (see Figure 7). In contrast, the worst experiences of participants included social distancing, the death of their loved ones, becoming infected by COVID-19, and being stuck at home all day (see Figure 8).

## 5. Discussion

In this study, we have analyzed the impact of COVID-19 on the lives of Bangladeshi students, focusing on the impact on their education, social life, daily life, plans, and mental health. Delayed education, disruptions to their social and daily lives, changes in plans or career paths, and financial difficulties among family members were some of the major concerns. Many participants now have now less focus on their studies and are spending more time on social media and online meetings, increasing their overall internet usage. As students are staying inside for safety reasons, they are communicating online more to stay connected with their friends and family, and this is contributing to a larger internet usage than before. Many also have changed and increased their sleep time and their personal care time. Since students do not need to visit classes physically and some of them do not have any online classes, they have less academic pressure now and thus spend plenty of time sleeping. As the students are spending more time at home, they now have the luxury of time to invest in their health, such as performing yoga/exercises or starting new hobbies. Many students have changed their plans. All of these factors have affected the mental health of the Bangladeshi students significantly. In total, 73.8% of students faced some form of depression during the pandemic and 78.2% faced mild to severe anxiety. Overall, female students suffered higher levels of depression and anxiety than male students.

The government of Bangladesh can use the outcomes of this study to implement measures to help alleviate the impacts of the pandemic on Bangladeshi students. Students should be given appropriate facilities for attending online classes. Proper guidelines are needed to maintain daily routines and social life. Measures can be taken to avoid excess usage of social media and other online social platforms. Overall internet usage needs to be confined within safe limits. The students should be encouraged to sleep according to recommended times, spend quality time with their parents, and take appropriate care of themselves. Many of the students have changed their career plans, and some of them can make good use of career counseling advice. More mental well-being centers or counseling services should be normalized, promoted, and highlighted in all educational institutions to help students to cope with the pandemic better. This study can be used for the further analysis of students’ mental health and to identify appropriate solutions to support students if similar cases or conditions reappear.

Our work has some limitations, such as working only with a small number of Bangladeshi students who are primarily pursuing undergraduate degrees. In future work, we will cover a larger proportion of the students (e.g., include school-level/college-level students), which we think will result in a huge variety of data that will give us bigger and deeper insights into the situations. If we can involve more students from a variety of backgrounds, we will be able to build solutions that will solve real-time problems in future. A long-term approach to this study can contribute to the field of behavioral pattern analysis of students. Different statistical and data mining techniques can be further applied to cluster students with different mental health conditions beforehand based on different associated factors. Thus, appropriate solutions can be implemented to ensure the overall well-being of the students in difficult times.

## Figures and Tables

**Table 1:** Demographic Characteristics of the Survey Participants (N = 409).

Demographic Characteristics	N (%)
Division	
Barisal	11 (2.7)
Chittagong	131 (32)
Dhaka	170 (41.6)
Khulna	20 (4.9)
Mymensingh	18 (4.4)
Rajshahi	30 (7.3)
Rangpur	24 (5.9)
Sylhet	5 (1.2)
Gender	
Female	151 (36.9)
Male	258 (63.1)
Age (year)	
18 to 24	294 (71.9)
25 to 34	114 (27.8)
More than 34	1 (0.2)
Education Level	
Bachelor student	389 (95.1)
Masters student	10 (2.4)
Recently completed graduation	10 (2.5)
Type of Educational Institution	
Public	126 (30.8)
Private	283 (69.2)
Subject/Discipline	
Engineering	323 (79)
Medical	26 (6.4)
Business Studies	17 (4.2)
Arts	11 (2.7)
Others	32 (7.8)

Here, N = Number of selected responses for analysis.

**Table 2:** Dependency analysis using chi-square test (N = 409). Here, *p* < 0.05 is considered as significant * and *p* < 0.001 is considered as highly significant **.

Factors	Depression		Anxiety	
	χ ${}^{2}$	*p*	χ ${}^{2}$	*p*
Factors in Education section				
Online Class	10.188	0.252	5.807	0.445
Facilities for Online Class	13.217	0.105	10.451	0.107
Study hour during pandemic	12.158	0.144	4.221	0.647
Focus during pandemic	50.477 **	0.000	26.426 *	0.009
Factors in Social Life section				
Time spent on social media	24.316 *	0.002	14.484 *	0.025
Time spent on online meetings	34.802	0.336	15.876	0.893
Time spent overall on internet	22.126 *	0.005	13.515 *	0.036
Factors in Daily Life section				
Sleep time	20.564 *	0.008	7.294	0.295
Time spent with family	12.905	0.115	10.546	0.103
personal care time	8.933	0.348	14.862 *	0.021
Family income Change	7.906	0.443	4.049	0.670
Factors in Future Plans section				
Change in future plans	17.489 *	0.002	19.966 **	0.000

## Data Availability

Data can be made available on reasonable request.
